# Structure of AQEE-30 of VGF Neuropeptide in Membrane-Mimicking Environments

**DOI:** 10.3390/ijms232213953

**Published:** 2022-11-12

**Authors:** One-Sung Park, Jeong-Kyu Bang, Chaejoon Cheong, Young-Ho Jeon

**Affiliations:** 1 College of Pharmacy, Korea University Sejong Campus, Sejong 30019, Korea; 2 Division of Bioconvergence Analysis, Korea Basic Science Institute, Cheongju 28119, Korea; 3 Department of Bioanalytical Sciences, University of Science and Technology, Cheongju 28119, Korea

**Keywords:** VGF, neuropeptide, HFIP, DPC

## Abstract

AQEE-30 is one of the VGF peptides, which are derived from the VGF polypeptide precursor, and related to various physiological phenomena including neuroprotective effects in Huntington′s disease and amyotrophic lateral sclerosis (ALS). Although various functions of AQEE-30 have been reported so far, the structure of this peptide has not been reported yet. In this study, the structure of human AQEE-30 was investigated in hexafluoroisopropanol (HFIP) and dodecyl phosphocholine (DPC) micelle solutions, using circular dichroism (CD) and nuclear magnetic resonance (NMR) spectroscopy. CD results showed that AQEE-30 had a partial helical structure in aqueous buffer, and the helical structure was stabilized in the HFIP and DPC micelle solutions. The 3D structures determined by NMR spectroscopy showed that AQEE-30 adopted mainly α-helical structure in both the HFIP and DPC micelle solutions. The surface of AQEE-30 showed that it was predominantly negatively charged. The residues from 601 to 611 in both the HFIP and DPC micelle solutions showed amphiphilicity with four negatively charged residues, glutamate. The C-terminal consecutive arginine residues formed a partial positively charged surface. These results suggest an α-helical active structure of AQEE-30 in the cell-membrane environment.

## 1. Introduction

**Figure 1:**
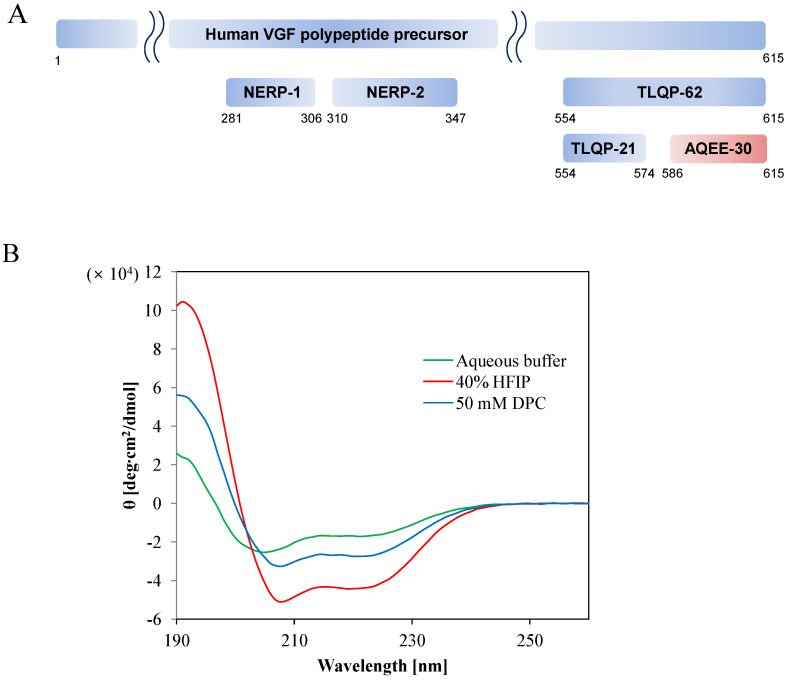
(**A**) Schematic representation of the human VGF polypeptide precursor and peptides. AQEE-30 is marked by reddish color. (**B**) Far-UV CD spectra of AQEE-30 at pH 7.4 in 40 % HFIP (red) and 50 mM DPC (blue) solutions. Far-UV CD spectra of AQEE-30 in aqueous buffer (green) at pH 7.4 were also measured as a control. In each measurement three scans were performed and averaged and backgrounds were subtracted.

AQEE-30 is a 30 amino acid peptide derived from VGF (non-acronym), a polypeptide precursor that is induced by various neurotrophins such as nerve growth factor, brain-derived neurotrophic factor, and neurotrophin-3 (Figure 1A) [1,2]. The VGF precursor is proteolytically processed by prohormone convertases PC1/3 and PC2 into various peptides [3,4]. Some VGF peptides have post-translational modifications such as phosphorylation, acetylation, sulfation, and amidation [5].

Not all VGF peptide activities are known but some bioactivities of VGF peptides including AQEE-30 have been reported. AQEE-30, AQEE-11, LQEQ-19, and HHPD-41 stimulate sympathetic outflow and promote penile erection in rats [6,7,8]. AQEE-30 and TLQP-62 regulate hippocampal electrical activity in vitro [2]. It is also known that AQEE-30 has anti-depressant activities and induces neurogenesis [9,10]. In STHdhQ111 cells in a Huntington′s disease model, AQEE-30 has a role in the suppression of cell death and the aggregation of mutant huntingtin [11]. AQEE-30 suppresses the loss of retinal ganglion cells (RGCs) after the optic nerve crush (ONC), increases the number of survival rat-derived RGCs, and promotes the outgrowth of neurites of rat and human iPSCs-derived RGCs in vitro [12]. AQEE-30 and LQEQ-19 have neuroprotective effects in an in vitro ALS model [13].

The VGF peptide level is differently regulated in various diseases or conditions. VGF mRNA is decreased in the Cornu Ammonis region of the hippocampus and Brodmann’s area 9 of the prefrontal cortex in human bipolar disorder [14]. VGF is reduced in the spinal cords of sporadic ALS patients [15]. In rats, AQEE-30 is significantly increased upon high caloric feeding [16]. After nerve injury and inflammation VGF level is rapidly upregulated and VGF-derived peptides including AQEE-30 evoke p38 activation in microglial cells and p38-dependent thermal hyperalgesia [17]. In mice, immunoreactive AQEE-30 was most abundant in the pituitary, while its brain levels were highest in the hypothalamus, striatum, and frontal cortex [18].

Several functions of AQEE-30 are reported but receptors are not identified, and the 3D structure of AQEE-30 remains unknown. Among VGF peptides there are two TLQP-21 receptors identified to date, and the structure of TLQP-21 upon a receptor binding also has been investigated [19,20,21]. TLQP-21 is disordered but adopts an α-helical conformation when targeting cells expressing complement-3a receptor1 (C3aR1), which is one of the known TLQP-21 receptors [21]. Previously we studied structures of Neuroendocrine regulatory peptide-2 (NERP-2), which is another VGF peptide, in the HFIP cosolvent and the DPC micelle solution [22]. The NERP-2 receptor is not identified either but we could determine the α-helical structure of NERP-2 in a membrane-mimicking environment using HFIP cosolvent and DPC micelle solution [22,23]. This result suggested that, even though receptors were not identified, we could expect the structures of peptides when the peptides bind to their receptors in the environment of cellular membranes. Likewise, here we investigated the structures of human AQEE-30 in HFIP and DPC micelle solutions. We used CD and NMR spectroscopy. AQEE-30 showed long α-helical structures along the sequence in the HFIP and DPC micelle solutions.

## 2. Results

### 2.1. Secondary Structure Studies Using CD Spectroscopy and a Helical Wheel Diagram

The secondary structural information of AQEE-30 was investigated by CD spectroscopy (Figure 1B). We used HFIP and DPC to provide a membrane-mimicking environment. HFIP is also known to stabilize the α-helical conformation of peptides [23]. In aqueous buffer, without HFIP or DPC micelle, the CD spectrum of AQEE-30 showed a minimum at 200 nm, a typical characteristic of random coil structure with partial helical structure. However, in the 40% HFIP and 50 mM DPC micelle solutions, the CD spectra showed two minima at 208 and 222 nm, typical characteristics of a helical structure [24,25,26,27,28,29]. The α-helical contents were estimated and averaged based on the CD spectra using three different algorithms (see Materials and Methods). In aqueous buffer, the α-helical content of AQEE-30 was 52.1%, while, when 40% HFIP and 50 mM DPC were added, the α-helical contents increased to 94.4 and 70.7%, respectively. This result indicated that the helical structure of AQEE-30 was more stabilized in the HFIP and DPC micelle solutions. In addition, the decreased random coil and increased helical contents of AQEE-30 in the HFIP cosolvent and DPC micelle solution implied that AQEE-30 might experience a conformational change upon membrane-receptor binding.

**Figure 2:**
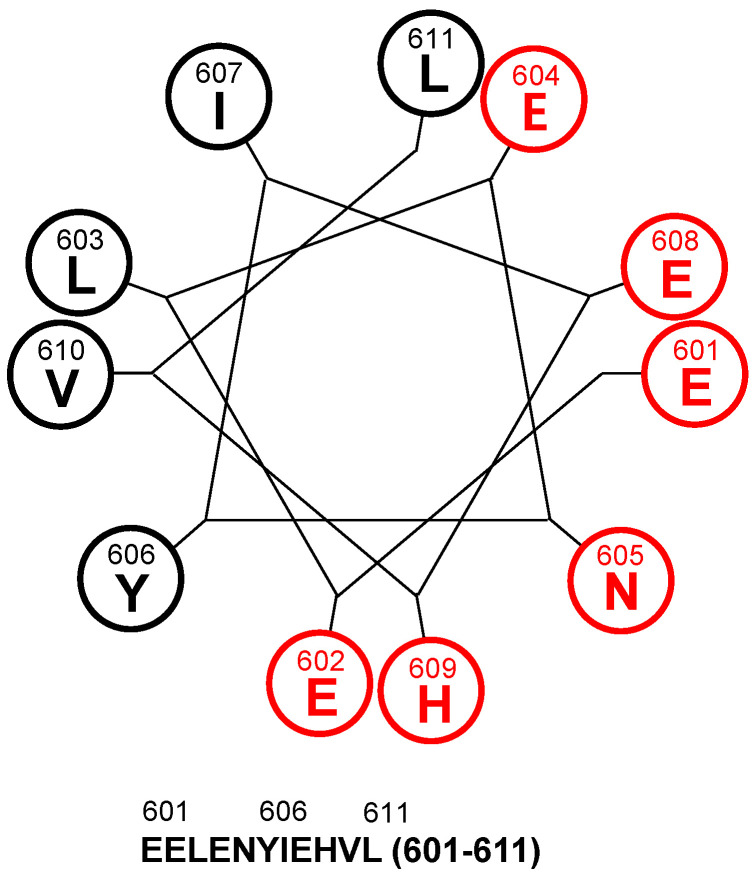
The helical wheel of AQEE-30. The helical wheel ranges from 601 Glu to 611 Leu, which shows amphiphilicity. The sequence is below the helical wheel. Hydrophilic residues are marked by red color and hydrophobic residues are marked by black color.

Because the CD spectra of AQEE-30 in the HFIP and DPC micelle solutions showed the possibility of AQEE-30 adopting a helical conformation upon membrane-receptor binding, we analyzed a helical wheel diagram of AQEE-30. The helical wheel showed amphiphilicity from Glu^601^ to Leu^611^ of AQEE-30 (Figure 2).

### 2.2. Resonance Assignments

To further investigate the three-dimensional structure of AQEE-30 in the HFIP and DPC micelle solutions, we determined the solution structures of the AQEE-30 peptide using NMR spectroscopy. Conventional triple-resonance NMR spectroscopy was used for sequence-specific assignments [30].

**Figure 3:**
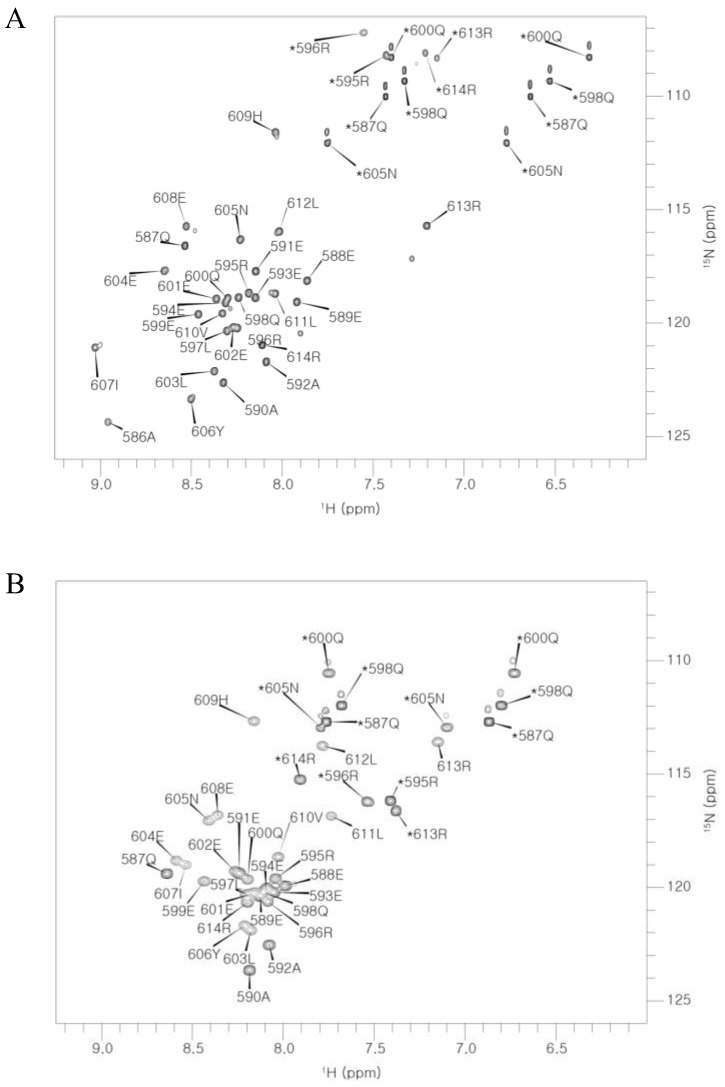
Assigned ^1^H-^15^N-HSQC spectra of AQEE-30 (**A**) in the HFIP solution and (**B**) in the DPC micelle solution. The assigned peaks are labelled with single letter amino acid names and sequence numbers. Side-chain peaks are marked by asterisks.

For the NMR experiments of the AQEE-30 peptide in the HFIP solution, the AQEE-30 peptide was labelled by nitrogen-15 and 40% HFIP was used as a cosolvent. With ^15^N-labelled AQEE-30 in the HFIP solution, ^15^N-TOCSY-HSQC and ^15^N-NOESY-HSQC spectra were obtained and used for the backbone and side-chain assignments (Figure 3A, Table 1). Additional HNHA and HNHB spectra were also obtained and used for the assignments. Of the 60 available backbone amide groups, 58 were assigned with nitrogen and hydrogen chemical shifts. A total of 163 of the 197 available hydrogens were assigned. Except for the backbone amide hydrogens, the rest of the hydrogens were assigned as either side chain or α-hydrogens (105 and 29, respectively). A total of 8 out of the 18 side-chain nitrogens were assigned.

Before NMR measurement of the AQEE-30 peptide in the DPC micelle solution, a series of CD spectra were obtained with a series of DPC concentrations from 50 mM to 2 mM to assess the appropriate minimum concentration of DPC for NMR experiments (Figure S1). The AQEE-30 concentration for CD experiments was set to 0.026 mM. From 50 mM down to 2 mM of DPC concentration, the AQEE-30 secondary structure gradually changed. In consideration of lower concentration and stable secondary structure, the DPC molar ratio to AQEE-30 for the NMR experiments was determined as 400:1. Accordingly, the AQEE-30 and DPC concentrations were set at 1 and 400 mM, respectively. For the NMR experiments of the AQEE-30 peptide in the DPC micelle solution, the AQEE-30 peptide was labelled by nitrogen-15 and carbon-13. With ^15^N- and ^13^C-labelled AQEE-30 in the DPC micelle solution, HNCACB, CBCA(CO)NH, (H)CC(CO)NH, HNHA, (H)CCH-TOCSY, HBHANH, and H(CCCO)NH spectra were obtained and used for the backbone and side-chain assignments (Figure 3B, Table 2). Of the 60 available backbone amide groups, 56 were assigned with nitrogen and hydrogen chemical shifts. Except for the backbone amide hydrogens, 157 hydrogens were assigned, with 129 assigned as side-chain hydrogens and 28 as α-hydrogens. A total of 8 out of the 18 side-chain nitrogens were assigned. Of the 158 available carbon atoms, 99 were assigned, with 69 assigned as side-chain carbons and 30 as α-carbons.

### 2.3. NMR Structures

**Figure 4:**
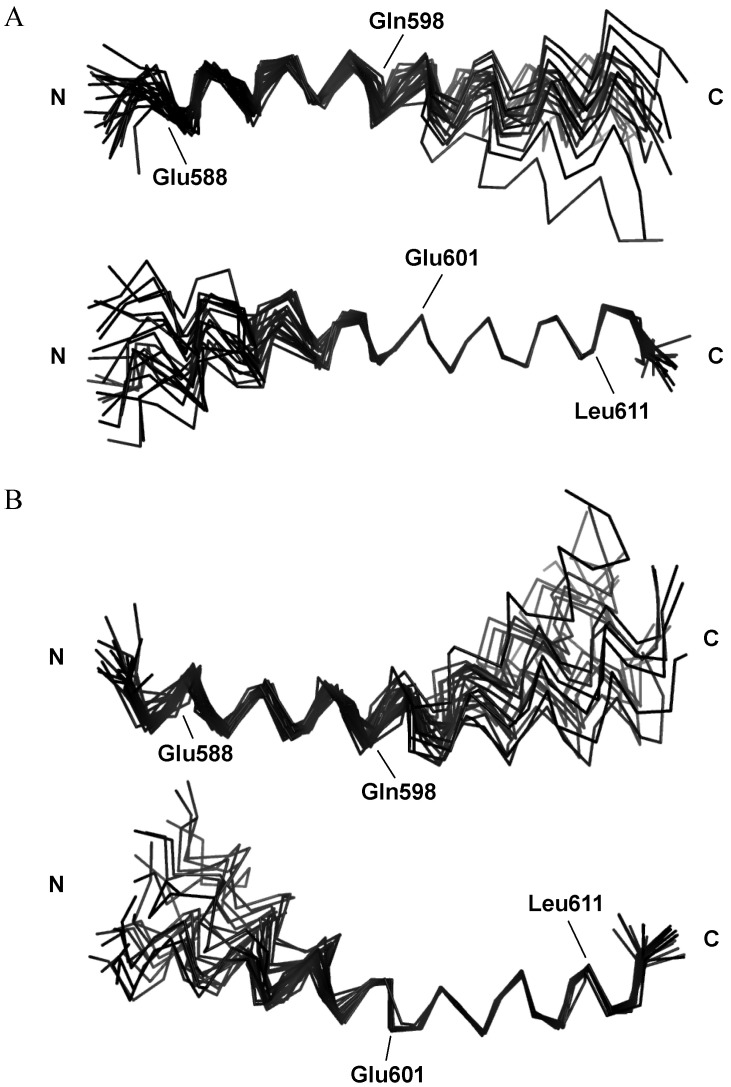
Conformations of AQEE-30 (**A**) in the HFIP solution and (**B**) in the DPC micelle solution. The structures have been solved with NMR spectra and the structure calculations were done using CYANA. Twenty models are represented for HFIP and DPC, respectively. Only the backbone is represented for clarity. The molecules were superimposed in the N-terminal region from 588 Glu to 598 Gln (upper side) and the C-terminal amphiphilic region from 601 Glu to 611 Leu (lower side) for HFIP and DPC, respectively. Boundary residues, 588 Glu and 598 Gln of the N-terminal region, 601 Glu and 611 Leu of the C-terminal amphiphilic region and termini are indicated.

The solution structures of AQEE-30 in each solution were determined with restraints derived from the NMR data (Figure 4, Table 3). A total of 54 dihedral angles were used as restraints to determine the AQEE-30 structures in the respective HFIP and DPC micelle solutions. ^15^N-NOESY-HSQC spectrum was used to calculate the distance restraints of the AQEE-30 peptide in the HFIP solution. The spectrum presented with 190 short-range restraints (|i − j| ≤ 1) and 99 medium-range restraints (1 < |i − j| < 5). Meanwhile, ^15^N-NOESY-HSQC and ^13^C-NOESY-HSQC spectra were used to calculate the distance restraints of the AQEE-30 peptide in the DPC micelle solution. The spectra presented with 178 short-range restraints (|i − j| ≤ 1), 61 medium-range restraints (1 < |i − j| < 5), and 3 long-range restraints (|i − j| ≥ 5). Percentages for the most favored Ramachandran regions were 95.2% and 94.0% for the HFIP and DPC micelle solutions, respectively.

In both the HFIP and DPC micelle solutions, AQEE-30 showed α-helical structures in most of the residues. When 20 models of AQEE-30 in the HFIP solution were aligned in the C-terminal region from Glu^601^ to Leu^611^, the backbone root-mean-square deviation (RMSD) value was 0.08, which demonstrates that this region converged well (Figure 4A). However, when aligned in the N-terminal region from Glu^588^ to Gln^598^, the backbone RMSD value was 0.90, showing less convergence. The backbone RMSD value from the full sequence was 1.61. Similarly, the ensemble of structures of AQEE-30 in the DPC micelle solution was converged well in the C-terminal region but showed less convergence in the N-terminal region. When 20 models of AQEE-30 in the DPC micelle solution were aligned in the C-terminal region from Glu^601^ to Leu^611^, the backbone RMSD value was 0.23 (Figure 4B). However, when aligned in the N-terminal region from Glu^588^ to Gln^598^, the backbone RMSD value was 0.76 and the backbone RMSD value from the full sequence was 1.72. The N-terminal region of AQEE-30 was flexible compared with the C-terminal region in both the HFIP and DPC micelle solutions. This result suggests that AQEE-30 can have a hinge region between the N-terminal and C-terminal regions when it adopts an α-helical structure.

## 3. Discussion

**Figure 5:**
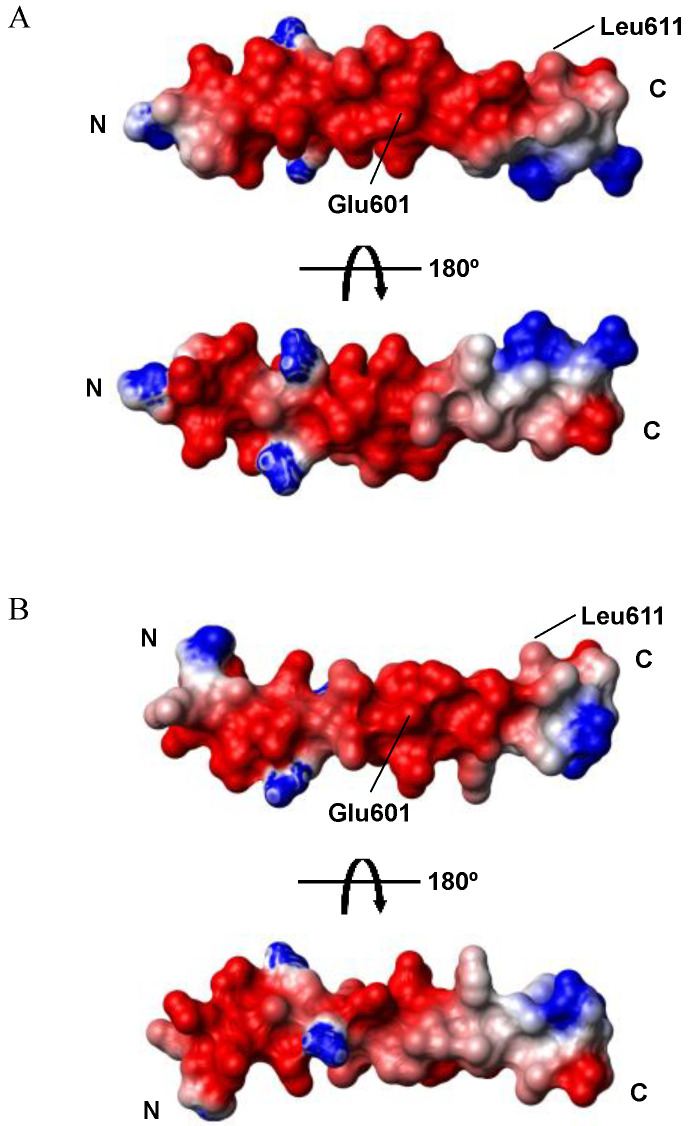
Electrostatic potential at the surface of AQEE-30 structures (**A**) in the HFIP solution and (**B**) in the DPC solution. Boundary residues, 601 Glu and 611 Leu of the amphiphilic region and termini are indicated.

The C-terminal region from Glu^601^ to Leu^611^ of AQEE-30 in HFIP and DPC micelle solutions showed relatively low RMSD values and converged well compared with the N-terminal region. The C-terminal region from Glu^601^ to Leu^611^ also showed amphiphilicity when we drew the helical wheel. Therefore, the difference in flexibility between the N-terminal region and the C-terminal region may be attributed to the hydrophobic part in the C-terminal region. Detergents like DPC provide a membrane-mimicking environment, and HFIP promotes a more ordered secondary structure. While detergents stabilize the structure of peptides by hydrophobic interactions, HFIP forms clusters around peptides and decreases the accessibility of the backbone hydrogen bonds to the water solvent, improving secondary structure stability [31,32]. The hydrophobic part in the C-terminal region of AQEE-30 may be stabilized in the DPC micelles by the interaction with the alkyl chains of the micelles. Conversely, the N-terminal region may be relatively flexible, interacting with the hydrophilic heads of the micelles and the water molecules. In the HFIP solution, the hydrophobic part in the C-terminal region of AQEE-30 also could interact with the hydrophobic parts of HFIP clusters around the AQEE-30 peptide, stabilizing itself. The surface electrostatic potentials of AQEE-30 in the HFIP and DPC micelle solutions were obtained using MOLMOL (Figure 5) [33]. Indeed, the surface electrostatic potentials showed the amphiphilic C-terminal region and the hydrophilic N-terminal region. Such amphiphilicity is a common feature of helical peptides for membrane binding [26,28,29,34]. The amphiphilic C-terminal region could lie in the interfacial part of cell membranes, with the long axis parallel to the cell-membrane plane. Therefore, the hydrophobic part of the amphiphilic C-terminal region could face and interact with the hydrophobic tails of cell membranes [29,35,36,37]. Conversely, from the CD data, in the HFIP solution the ellipticity θ_222_ was less than −33,000, which is the normal value for a single α-helix. This result suggests a possibility that, upon membrane binding, the helices of AQEE-30 form bundles with the hydrophilic parts facing each other and the hydrophobic parts interacting with the hydrophobic tails of cell membranes, and that such bundles could be in a perpendicular orientation to the cell-membrane plane [38,39]. However, the exact mechanism of HFIP stabilizing the helical structure is not known yet, and in the DPC micelle solution the ellipticity θ_222_ was in a normal range of value for a single α-helix. Thus, the membrane-bound orientation of AQEE-30 remains an open question.

Many α-helices in proteins are gently bent but helices in coiled-coil proteins are more linear than those in globular proteins [39,40]. Internal helices are more closely aligned with each other than those on the surface, in which the carbonyl oxygen atoms form additional hydrogen bonds with the solvent [39,40,41,42,43]. Such additional hydrogen bonds with the solvent are related to the longer and less linear hydrogen bonds, and they can cause changes in helix main-chain torsion angles and therefore lead to helix curvature [39,40,41]. As shown in Figure 4, AQEE-30 helices seem to be bent in both the HFIP and DPC micelle solutions. However, the AQEE-30 structures in the HFIP solution seem to be less bent than those in the DPC micelle solution. The helices of AQEE-30 in the HFIP solution can form bundles, and helices in the coiled-coil proteins are more linear [39,40]. Therefore, less bending of the AQEE-30 structures in the HFIP solution than those in the DPC micelle solution seems to be reasonable.

Although receptors of AQEE-30 are not identified yet, our investigation suggests that AQEE-30 may experience conformational changes upon membrane binding, and such conformational changes may help to bind receptors. Receptors of TLQP-21, another VGF peptide, have been identified, and the structure of the receptor-bound state of TLQP-21 has been investigated [19,20,21]. TLQP-21 showed a disordered conformation but adopted an α-helical conformation when targeting cells expressing complement-3a receptor1 (C3aR1), which is one of the TLQP-21 receptors [21]. Neurotensin, another neuropeptide, also showed conformational changes in HFIP and DPC micelle solutions, and the structure of neurotensin in an HFIP solution was closer to the structure of the receptor-bound state [44,45]. From the structures of AQEE-30 in the HFIP and DPC micelle solutions, which promoted a more ordered secondary structure and provided a membrane-mimicking environment, respectively, we can deduce that AQEE-30 may change the conformation when it binds cell membranes. The amphiphilicity of the helical structure of AQEE-30 may help to bind cell membranes and form the helical structure. According to the two-step ligand transportation model, ligands bind cell membranes and then diffuse through the cell membranes two-dimensionally to search for receptors [46]. Once the AQEE-30 peptide binds cell membranes, it will find receptors diffusing through the cell membranes and, when it meets the receptors, the α-helical structure of the AQEE-30 peptide induced upon membrane binding may enhance receptor binding.

## 4. Materials and Methods

### 4.1. Peptide Expression and Purification

The cDNA coding for human VGF was synthesized (Cosmogenetech, Seoul, Korea). Human AQEE-30 (residues 586 to 615) was amplified by a polymerase chain reaction (PCR). The amplified AQEE-30 DNA was cloned into a modified pET28a vector, which included the small ubiquitin-like modifier (*SUMO*) gene between the NheI and BamHI enzyme sites. The cloned plasmid encoded the AQEE-30 peptide with a His-SUMO tag attached at the N-terminal. The plasmid was transformed into *E. coli* Rosetta (DE3) competent cells. The cells were cultured in LB media for CD spectroscopy and M9 media supplemented with ^15^N-ammonium chloride and ^13^C-glucose or with ^15^N-ammonium chloride only for NMR spectroscopy. Overexpression of AQEE-30 was induced by 1 mM isopropyl-β-d-1-thiogalactopyranoside (IPTG). After the addition of IPTG, the culture was incubated for about 3 h at 37 °C. The cells were harvested and resuspended in lysis buffer (50 mM Tris, 100 mM NaCl, 1 mM phenylmethylsulfonyl fluoride (PMSF), pH 8.0) and then lysed by sonication. The cell lysates were centrifuged for 90 min at 35,860× *g*. The AQEE-30 in the supernatant was then purified by a series of chromatographic methods. Ni^2+^-affinity chromatography was performed using a HisTrap column (GE Healthcare Life Sciences, Chicago, IL, USA) with an appropriate binding (50 mM Tris, 100 mM NaCl, pH 8.0) and elution buffer (50 mM Tris, 50 mM NaCl, 500 mM imidazole, pH 8.0). The eluted SUMO-attached fusion peptides were then treated with Ulp1 at 4 °C overnight to cleave the His-SUMO tags. Then, an ion-exchange chromatography was performed using a HiTrap Q column (GE Healthcare Life Sciences, Chicago, IL, USA) with an appropriate binding (50 mM Tris, pH 8.0) and elution buffer (50 mM Tris and 1 M NaCl, pH 8.0). Ni^2+^-affinity chromatography was performed again to remove the Ni^2+^ bead-binding proteins including the detached His-SUMO tag. Finally, size-exclusion chromatography was performed using a HiLoad 16/600 Superdex 75 prep-grade column (GE Healthcare Life Sciences, Chicago, IL, USA). The final buffer used in the size-exclusion chromatography step was 50 mM Tris (pH 7.4) with 50 mM NaCl. The purified AQEE-30 peptide had an additional Ser at the N-terminal as a cloning artifact.

### 4.2. CD Spectroscopy

Buffer components for CD spectroscopy samples were 25 mM potassium phosphate (pH 7.4), and 50 mM sodium sulfate. 40% HFIP (Sigma-Aldrich, St. Louis, MO, USA) or 50 mM DPC (Affymetrix Anatrace Products, Maumee, OH, USA) was used. The AQEE-30 peptide concentration was 0.1 mg/mL for all the samples. CD spectra were measured in a wavelength range of 190–260 nm on a JASCO J-710 spectropolarimeter (JASCO Corporation, Tokyo, Japan) with a 1-mm path quartz cuvette. Every measurement was performed three times and averaged, and then the background was subtracted. The contents of secondary structures were estimated from the CD spectra using three computer programs, SELCON3, CDSSTR, and CONTIN/LL, and averaged [47,48,49,50].

### 4.3. NMR Spectroscopy

For the HFIP sample, the ^15^N-labelled AQEE-30 peptide was prepared. The concentration of ^15^N-labelled AQEE-30 was 1 mM and the HFIP concentration was 40%. For the DPC sample, ^15^N- and ^13^C-labelled AQEE-30 peptide was prepared. The concentration of ^15^N- and ^13^C-labelled AQEE-30 was 1 mM and the DPC concentration was 400 mM. For both samples, 25 mM sodium phosphate (pH 6.0) was used as a buffer without any other salt components, and the deuterium oxide (D_2_O) concentration was 10%.

For the HFIP sample, 2D ^15^N-HSQC and 3D HNHA, HNHB, ^15^N-TOCSY-HSQC, and ^15^N-NOESY-HSQC experiments were carried out at 25 °C on a Bruker NMR instrumentwith a proton frequency of 900 MHz (Bruker Corporation, Billerica, MA, USA). For the DPC sample, 2D ^15^N-HSQC and ^13^C-HSQC, 3D HNCACB, CBCA(CO)NH, (H)CC(CO)NH, HNHA, (H)CCH-TOCSY, HBHANH, H(CCCO)NH, ^15^N-NOESY-HSQC and ^13^C-NOESY-HSQC were carried out at 15 °C on a Bruker NMR instrument with a proton frequency of 700 MHz (Bruker Corporation, Billerica, MA, USA). The pulse programs were obtained from the Bruker standard pulse program library. Water suppression was done using coherent selection by the pulsed-field gradient. All the NMR data were processed using TopSpin. Processed spectra were visualized and analyzed using the CcpNmr Analysis software [51]. Assignment of the backbone and side-chain NMR resonances was done with standard assignment protocols.

### 4.4. Structure Calculations

The structures of AQEE-30 in the HFIP and DPC micelle solutions were calculated using the CYANA software based on the restraints obtained from the NMR data and analyses [52]. Dihedral angle restraints were obtained from the chemical shifts using DANGLE [53]. Distance restraints were obtained from the 3D NOESY-HSQC spectra. Cross peaks in the NOESY-HSQC spectra were manually picked and automatically assigned during the structure calculation by CYANA. The structures were calculated with 10,000 torsion angle dynamics steps and 1 Å violation tolerances. The calculation produced 100 initial structures and 20 final structures.

## 5. Conclusions

We investigated the structures of AQEE-30 in HFIP and DPC micelle solutions, which promoted a more ordered secondary structure and provided a membrane-mimicking environment, respectively, using CD and NMR spectroscopy. AQEE-30 showed conformational changes, increasing α-helical content in the HFIP and DPC micelle solutions. This suggests that AQEE-30 may experience conformational changes upon membrane binding. Amphiphilicity of the helical structure of AQEE-30 may facilitate such conformational changes. In addition, conformational changes may promote receptor binding. This structural information may help further studies of AQEE-30 for mechanisms of receptor binding and functions.

## Figures and Tables

**Table 1:** AQEE-30 chemical shifts of the assigned 1H resonances in the HFIP solution.

	HN	Hα	Hβ	Others
Ala586	8.96	4.27	1.55	-
Gln587	8.53	4.09	2.20, 2.05	Hγ 2.48; Hε 6.63, 7.43
Glu588	7.86	4.15	2.15, 2.29	Hγ 2.39, 2.47
Glu589	7.92	4.15	2.22	Hγ 2.44, 2.56
Ala590	8.32	4.23	1.59	-
Glu591	8.15	4.10	2.25, 2.18	Hγ 2.38, 2.63
Ala592	8.09	4.17	1.63	-
Glu593	8.15	4.29	2.22, 2.25	Hγ 2.49
Glu594	8.31	4.09	2.27	Hγ 2.41, 2.57
Arg595	8.18	4.18	2.07	Hγ 1.78, 1.88; Hδ 3.30; Hε 7.43; Hη 6.64
Arg596	8.25	4.17	2.11	Hγ 1.74, 1.89; Hδ 3.24, 3.34; Hε 7.55; Hη 6.71
Leu597	8.30	4.20	1.73, 2.00	-
Gln598	8.24	4.14	2.38, 2.28	Hγ 2.49, 2.69; Hε 6.53, 7.33
Glu599	8.46	4.12	2.37, 2.20	Hγ 2.65
Gln600	8.30	4.09	2.34	Hγ 2.46, 2.58; Hε 6.31, 7.40
Glu601	8.36	4.16	2.29	Hγ 2.46, 2.61
Glu602	8.27	4.20	2.33, 2.25	Hγ 2.46, 2.58
Leu603	8.37	4.34	2.03, 1.81	-
Glu604	8.65	4.00	2.24	-
Asn605	8.23	4.60	3.15, 2.94	Hδ 6.77, 7.75
Tyr606	8.50	4.33	3.49, 3.35	-
Ile607	9.03	3.58	2.05	-
Glu608	8.53	3.90	2.15, 1.86	-
His609	8.04	4.61	3.33	-
Val610	8.33	3.89	1.93	Hγ 0.85
Leu611	8.04	4.25	1.64, 1.96	-
Leu612	8.02	4.38	2.00, 1.59	-
Arg613	7.20	4.20	1.95	Hγ 1.66; Hδ 3.24; Hε 7.16
Arg614	8.11	4.70	1.95	Hγ 1.77; Hδ 3.23; Hε 7.22
Pro615	-	-	-	-

**Table 2:** AQEE-30 chemical shifts of the assigned 1H resonances in the DPC micelle solution.

	HN	Hα	Hβ	Others
Ala586	-	4.21	1.42	-
Gln587	8.64	4.13	2.07, 1.94	Hγ 2.35; Hε 7.76, 6.87
Glu588	7.99	4.09	2.05	Hγ 2.27
Glu589	8.13	4.10	2.05	Hγ 2.37, 2.28
Ala590	8.18	4.21	1.44	-
Glu591	8.24	4.06	2.04	Hγ 2.24, 2.44
Ala592	8.08	4.11	1.48	-
Glu593	8.05	4.25	2.06	Hγ 2.30
Glu594	8.09	4.06	2.06	Hγ 2.36
Arg595	8.04	4.10	1.89	Hγ 1.55; Hδ 3.21; Hε 7.41
Arg596	8.09	4.10	1.90	Hγ 1.72; Hδ 3.13; Hε 7.54
Leu597	8.20	4.08	1.82, 1.58	Hδ 0.84, 0.90
Gln598	8.11	4.09	2.16	Hγ 2.38, 2.51; Hε 7.68, 6.80
Glu599	8.44	4.06	1.99, 2.17	Hγ 2.48
Gln600	8.20	4.04	2.16	Hγ 2.32; Hε 6.73, 7.75
Glu601	8.16	4.08	2.10	Hγ 2.38
Glu602	8.26	4.08	2.08	Hγ 2.45, 2.28
Leu603	8.18	4.19	1.77, 1.90	Hγ 1.71; Hδ 0.99, 0.93
Glu604	8.59	3.81	2.17	Hγ 2.52
Asn605	8.41	4.42	2.96, 2.84	Hδ 7.79, 7.10
Tyr606	8.21	4.25	3.25	-
Ile607	8.54	3.49	2.00	Hγ 0.88,1.15; Hδ 0.88
Glu608	8.36	3.76	1.92, 1.77	Hγ 2.22
His609	8.16	4.64	3.26	-
Val610	8.03	3.81	1.80	Hγ 0.78, 0.82
Leu611	7.74	4.15	1.52, 1.87	Hδ 0.92, 0.84
Leu612	7.78	4.27	1.52, 1.98	Hγ 1.62; Hδ 0.80, 0.83
Arg613	7.15	4.02	1.90, 1.82	Hγ 1.53; Hδ 3.13; Hε 7.38
Arg614	8.20	4.50	1.87, 1.57	Hγ 1.67; Hδ 3.13; Hε 7.91
Pro615	-	4.21	1.89, 2.20	Hγ 1.96; Hδ 3.77, 3.64

**Table 3:** NMR structural statistics.

	HFIP	DPC
dihedral angles		
PHI	27	27
PSI	27	27
distance restraints		
short-range NOEs, |i − j| ≤ 1	190	178
medium-range NOEs, 1 < |i − j| < 5	99	61
long-range NOEs, |i − j| ≥ 5	0	3
total	289	242
average target function value	0.12	0.29
rmsd analysis (Å)		
E601-L611 backbone	0.08	0.23
restraints violated in 6 or more structures		
violated distance restraints	2	1
violated van der Waals restraints	0	0
violated angle restraints	0	0
Ramachandran regions (%)		
most favored	95.2	94.0
additionally allowed	4.5	5.7
generously allowed	0.0	0.0
disallowed	0.3	0.3

## Data Availability

The structures were deposited to Protein Data Bank as PDB ID 8H0G and 8H0U, which correspond to the AQEE-30 structures in HFIP solution and DPC micelle, respectively. NMR chemical shift data were deposited to Biological Magnetic Resonance Bank as BMRB ID 36512 and 36513, which correspond to the AQEE-30 signals in HFIP solution and DPC micelle, respectively.

## Supplementary Materials

The supporting information can be downloaded at: https://www.mdpi.com/article/10.3390/ijms232213953/s1.
