# Supplementary material for: Structure of AQEE-30 of VGF Neuropeptide in Membrane-Mimicking Environments
Source: Int J Mol Sci. 2022 Nov 12;23(22):13953. doi: 10.3390/ijms232213953 (PMC9696787; doi:10.3390/ijms232213953)
Supplement: Supplementary file 1 [file ijms-23-13953-s001.zip › ijms-1997166-supplementary.pdf]

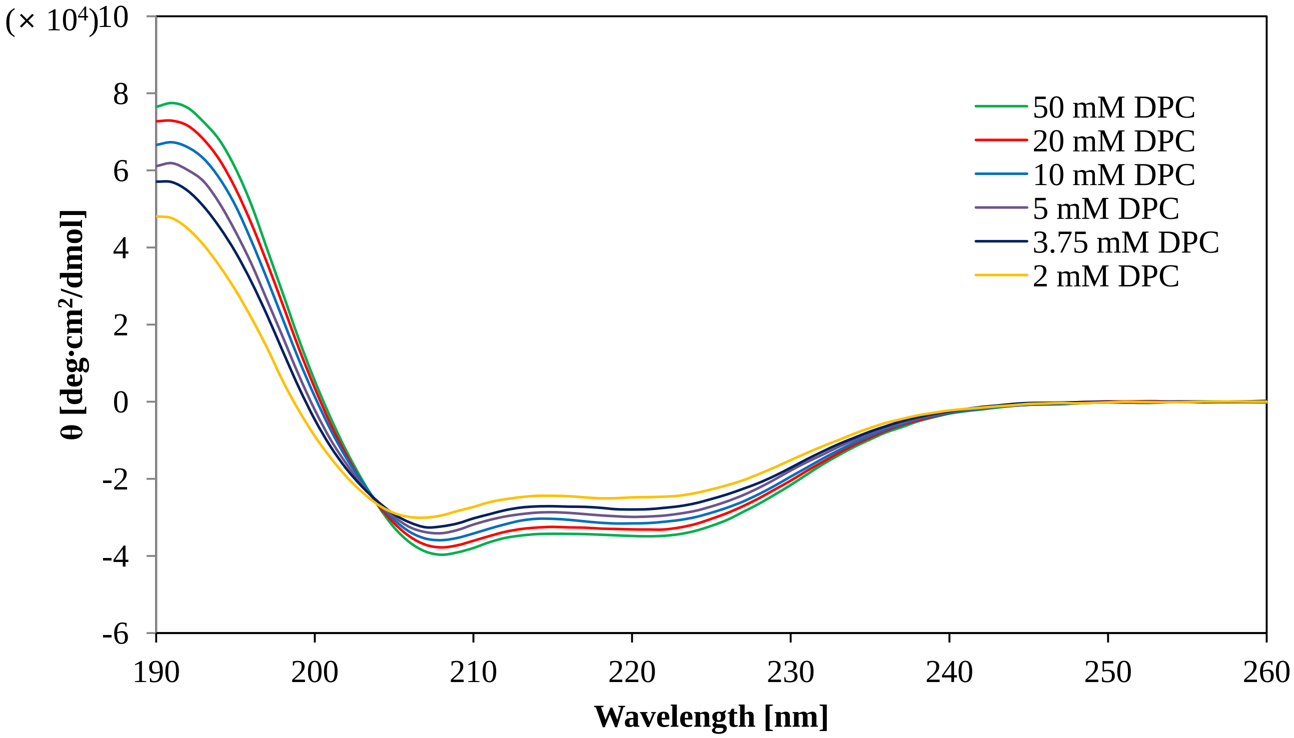

Figure S1: Far-UV CD spectra of AQEE-30 at pH 7.4 in the 50 mM (green), 20 mM (red), 10 mM (blue), 5 mM (purple), 3.75 mM (navy), and 2 mM (orange) DPC solutions. In each measurement three scans were performed and averaged and backgrounds were subtracted.
